# Display of recombinant proteins at the surface of lactic acid bacteria: strategies and applications

**DOI:** 10.1186/s12934-016-0468-9

**Published:** 2016-05-03

**Authors:** C. Michon, P. Langella, V. G. H. Eijsink, G. Mathiesen, J. M. Chatel

**Affiliations:** Micalis Institute, INRA, AgroParisTech, Université Paris-Saclay, 78350 Jouy-en-Josas, France; Department of Chemistry, Biotechnology and Food Science, Norwegian University of Life Sciences (NMBU), Ås, Norway

**Keywords:** Lactic acid bacteria, Genetic engineering, Surface display

## Abstract

Lactic acid bacteria (LAB) are promising vectors of choice to deliver active molecules to mucosal tissues. They are recognized as safe by the World Health Organization and some strains have probiotic properties. The wide range of potential applications of LAB-driven mucosal delivery includes control of inflammatory bowel disease, vaccine delivery, and management of auto-immune diseases. Because of this potential, strategies for the display of proteins at the surface of LAB are gaining interest. To display a protein at the surface of LAB, a signal peptide and an anchor domain are necessary. The recombinant protein can be attached to the membrane layer, using a transmembrane anchor or a lipoprotein-anchor, or to the cell wall, by a covalent link using sortase mediated anchoring via the LPXTG motif, or by non-covalent liaisons employing binding domains such as LysM or WxL. Both the stability and functionality of the displayed proteins will be affected by the kind of anchor used. The most commonly surfaced exposed recombinant proteins produced in LAB are antigens and antibodies and the most commonly used LAB are lactococci and lactobacilli. Although it is not necessarily so that surface-display is the preferred localization in all cases, it has been shown that for certain applications, such as delivery of the human papillomavirus E7 antigen, surface-display elicits better biological responses, compared to cytosolic expression or secretion. Recent developments include the display of peptides and proteins targeting host cell receptors, for the purpose of enhancing the interactions between LAB and host. Surface-display technologies have other potential applications, such as degradation of biomass, which is of importance for some potential industrial applications of LAB.

## Background

Lactic acid bacteria (LAB) have been used for thousands of years in food products where their fermentative properties promote natural conservation and taste development [1]. LAB are a heterogeneous group of gram-positive bacteria, including species of *Lactococcus, Lactobacillus* and *Streptococcus*, which during fermentation of carbohydrates produce lactic acid as a major end product. The majority of LAB strains are capable of surviving the harsh conditions in the gastro-intestinal tract (GIT), and some are capable of colonizing certain intestinal tissues. It has to be noticed that *Lactococcus lactis*, the most used LAB for heterologous protein expression, only survive for a few hours in the human GIT [2] whereas survival times for some lactobacilli are much higher until 7 days. This physiological characteristic could be an asset for *L. lactis* as genetically modified organism (GMO) because, as the bacterium dies in the GIT, it cannot be spread in the environment. Since LAB have been consumed for ages and are commonly found in food products they are considered as safe by the World Health Organization. Several LAB, and especially members of the genus *Lactobacillus,* have health promoting properties. Strong resistance to harsh conditions, innocuous activity and beneficial properties make LAB excellent candidates to deliver active molecules such as vaccines or cytokines to the GIT mucosa [3].

Over the last 20 years, wide and numerous health applications of LAB have been demonstrated. For example, a *L. lactis* strain producing recombinant Elafin, a protease inhibitor, has been shown to have a positive effect on gluten-related disorders and in the control of inflammatory bowel disease in an induced colitis model [4, 5]. LAB have also been used to produce medically interesting proteins like tetanus toxin [6, 7], insulin [8] or leptin [9], to deliver cDNA to eukaryotic cells [10] and several recombinant LAB have entered clinical studies [11, 12].

Recombinant protein can be produced in the cytoplasm, end up in the cell membrane or be exported from the cells to end up in the surroundings (secreted) or to become anchored at the bacterial surface. Secreted proteins are diluted in the environment and are likely to be prone to proteolytic degradation as well as effects of low pH and bile salts, all of which may weaken the activity and functionality of the protein [13, 14]. To protect the protein from harsh conditions and to increase its on-site concentration, strategies based on anchoring the protein to the microbial surface are gaining increasing attention. It is conceivable that a protein attached to the bacterial surface, is more protected than a free protein, especially if the protein is embedded in the bacterial cell wall.

In this review we describe the diverse techniques to display functional proteins at LAB surfaces, review comparative studies of strains directing the same protein to different locations, and discuss specific advantages of protein surface display.

## Strategies to display proteins at the surface of lactic acid bacteria

**Fig. 1:**
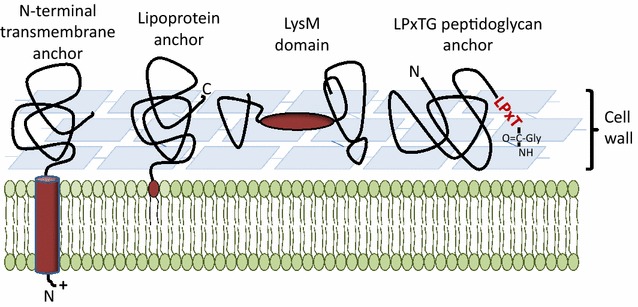
Methods for protein display in lactobacilli. A schematic view of the most exploited anchoring methods that are based on covalent or non-covalent interactions with components of the cell membrane or the cell wall. *Dark red* shows anchor domains/motifs, which are coupled to the to-be-displayed protein. The various routes for display are discussed in the main text. Additional variants based on using a binding domain (i.e. similar to the LysM domain-based strategy) are also discussed in the text. Note that the LysM domain may have various positions relative to the to-be expressed protein; see text

The first heterologous bacterial display system was constructed almost 30 years ago [15, 16]. In these early studies, short, heterologous gene fragments were inserted into genes encoding outer membrane proteins of *Escherichia coli* leading to peptide display at the bacterial surface. It was suggested that surface display would open up for development of oral live vaccines using non-pathogenic strains [15]. Later on, several systems have been established for surface-anchoring and displaying heterologous proteins, including systems for gram-positive bacteria [17, 18]. Gram-positive bacteria have only one cytoplasmic cell membrane, meaning that protein export only involves one membrane translocation. Both the single cell membrane and the relatively thick cell wall of Gram-positive bacteria offer anchoring opportunities. There are two principally different ways to anchor secreted proteins to the bacterial surface, by covalent binding to surface components or by non-covalent binding to the cell membrane or cell wall components through specialized binding domains [19] (Fig. 1).

Display of proteins implies a trade-off between high exposure, which will improve interactions with the GIT, and a low exposure, which will protect the displayed protein. Proteins displayed as compact properly folded molecules are likely to be less susceptible to degradation than engineered fusion proteins such as multi-epitope antigen fusions. Exposure of the protein will depend on the type of anchor used, although the degree of exposure and its effect on e.g. immunogenicity cannot be measured or rationalized. Fact is that the use of different anchoring systems can result in distinctly different host responses [20, 21]; this should be taken into consideration when designing LAB-based delivery strategies.

Expression and export of a heterologous protein containing the desired anchoring signal is at the basis of most display strategies. The success of these steps is a major determinant of both the viability of the recombinant strain and the amount of protein displayed on its surface. Assuming that the primary gene product is produced at desirable levels, its proper secretion and post-translational processing are by no means assured. Insufficiently effective secretion may lead to overloading of the secretion machinery and secretion stress, which may cause reduced growth of the host and/or promote intracellular or extracellular proteolytic degradation [22, 23]. Unfortunately, the secretion efficiency for heterologous proteins is difficult to predict. It depends not only on the signal peptide, but also on an optimal combination between the signal peptide and the target protein and, most likely, the interplay between expression rate, folding rate and secretion rate [23–27]. Since it is not possible to predict which signal peptide works best for a certain protein, it is generally advisable to test several to find the most optimal one, possibly by evaluating genome-wide signal peptide libraries in modular cloning systems (see below). In early work in the field, Dieye et al. [27] showed that replacement of the M6 signal peptide with the Usp45 signal peptide from *L. lactis* doubled the amount of a staphylococcal nuclease reporter protein (NucA) that was successfully anchored to the cell wall [27]. It has also been shown that insertion of a negatively charge synthetic pro-peptide immediately downstream of the signal peptidase cleavage site increases secretion efficiency [28]. The optimization of secretion efficiency and the reduction of secretion stress are of utmost importance when the aim is surface display. Notably, several of the recombinant strains appearing in the examples discussed below showed impaired growth, meaning that further optimization, e.g. by adjusting expression levels and/or secretion and anchoring signals, may be useful.

In LAB, expression, secretion and surface display of heterologous target proteins have been explored quite extensively [27, 29–37]. The nisin-based NICE system is a well-known system for inducible expression in *L. lactis* and several other LAB [32]. A system similar to the NICE system and primarily developed for use in lactobacilli is the pSIP system [38, 39] In recent years, this modular vector system for inducible gene expression has been developed for protein secretion and anchoring [21, 23, 40–44], exploiting signals from *L. plantarum* WCFS1.

Below, the varying anchoring strategies displayed in Fig. 1 are discussed in detail and references illustrating the use of these strategies are provided. All these strategies have to some extent been implemented in the pSIP system.

### N-terminal transmembrane anchors

Many secreted proteins have a Sec-type N-terminal signal peptide [45] which is recognized intracellularly and guided to a translocation machinery that transports proteins over the cell membrane in an unfolded state. After or during translocation, the signal peptide is cleaved off and the mature protein is released into the surroundings, ending up in the culture medium or remaining associated to the cells by covalent anchoring or looser forms of association (Fig. 1) [46]. Some proteins with a secretory signal sequence lack the signal peptidase cleavage site. In these proteins, the signal peptide (SP), with its central stretch of hydrophobic residues, acts as an N-terminal transmembrane helix anchoring the protein to the membrane. Whereas the absence of a signal peptidase cleavage site strongly influences the localization of the protein, it is not straightforward to predict with certainty which signal peptides are cleaved off and which are not [47].

To achieve N-terminal transmembrane anchoring, the protein of interest needs to be translationally fused to the anchoring sequence and the length of the latter needs careful consideration. One may vary from using very short anchors, comprising the SP followed by a few additional residues functioning as linker, to fusing the protein of interest to a complete N-terminally anchored protein. A short anchor may leave the target protein totally embedded in the cell wall, which may lead to protection but also to lack of accessibility. In early work based on randomly screening export-signals from a genomic library of *L. lactis* and using the *Staphylococcus aureus* nuclease as reporter protein, Poquet et al. [48] detected two anchors predicted to contain a single N-terminal transmembrane helix, varying in size from 40 to 234 residues. One of the most exploited N-terminal transmembrane helix anchors is the PgsA protein from *Bacillus subtilis*, which is part of the poly-γ-glutamate synthetase complex. Several reports describe coupling of antigens to the C-terminus of PgsA, leading to successful surface display in *L. casei* and *L. lactis* [12, 33, 49–54]. In these studies, surface-display was confirmed using fluorescence microscopy or flow cytometry. For some of the engineered strains specific immune responses in animal models, mostly mice, could be demonstrated, indicating that this type of anchoring gives proper localization, stability and accessibility of the antigens [49, 50].

### Lipoprotein-anchors

Lipoproteins contain an N-terminal signal peptide with a so-called lipobox motif in the C-terminal part of the signal peptide [55, 56]. After secretion via the Sec-pathway, diacylglycerol transferase catalyzes a coupling reaction between a conserved cysteine in the lipobox and a phospholipid of the membrane. The SP is then cleaved off by a lipobox specific signal peptidase, SPase II. The modified cysteine now forms the N-terminus of the mature lipoprotein which is covalently bound to a phosholipid of the cell membrane [56, 57]. Fusing a heterologous protein sequence to a lipoprotein downstream of the lipobox is thus a method for membrane anchoring and surface display.

In LAB, the exploitation of lipoproteins as anchor for heterologous proteins has received little attention compared to other anchoring methods. In their screening study with the NucA reporter (see above) Poquet et al. [48] found four *Lactococcus* lipoprotein fragments that served as surface anchors, varying in length from 91 to 140 residues (note that the actual N-terminal signal peptides are about 25 residues).

Successful anchoring and display in *L. lactis* has also been achieved using the lactococcal basic membrane protein A (BmpA) [58, 59]. Interestingly, reduction of the length of the BmpA anchor from full length (352 residues) to versions shortened to 44–104 residues led to increased detection of the reporter protein (the B-domain of staphylococcal protein A) on the bacterial surface [59]. This indicates that not only anchor length, but also other anchor features such as the orientation of the C-terminus (and, thus, of the displayed protein) play a role in determining the overall efficiency of the display system.

In *Lactobacillus,* fragments of the 547 residue oligopeptide ABC transporter (Lp_1261) and the 298 residue peptidylprolyl isomerase PrsA (Lp_1452) have been used successfully as lipo-anchors in order to display a protein responsible for invasivity of *Yersinia pseudotuberculosis* called invasin [21]. The lengths of the anchor sequences were 75 and 142 residues, respectively, including the signal peptide (22 and 19 residues, respectively) and regions N-terminal of the catalytic domains of these proteins as they were predicted by Pfam. In both cases, surface display of both the complete extracellular part of invasin (domains D1-D5) and the D4-D5 fragment was demonstrated. Surprisingly, the lipo-anchored proteins were not evenly detected over the cell surface, but appeared mainly in and near the septum. For other cell wall anchors explored in the same study (an N-terminal transmembrane anchor, see above, and a LysM anchor, see below) such an asymmetric distribution was not observed. Recombinant strains expressing lipo-anchored full length invasin induced a strong pro-inflammatory response in monocytes similar to the response induced by the positive control, bacterial lipopolysaccharides (LPS) [21].

### Covalent anchoring to the cell wall through LPXTG anchors

**Fig. 2:**
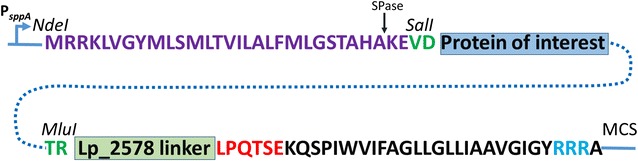
Covalent anchoring to the lactobacillal cell wall. The picture shows a schematic overview of the expression cassette for cell wall anchoring of a protein of interest, using an anchoring sequence derived from the Lp_2578 protein of *L. plantarum*. The cassette is translationally fused to the inducible PsppA promoter and all parts are easily interchangeable using introduced restriction sites (*Nde*I, *Sal*I, *Mlu*I and multiple cloning site, MCS). The primary gene product comprises a signal peptide (indigo), which in this example is derived from Lp_2578, but which could be any peptide from a signal peptide library [23]. The predicted signal peptide cleavage site is indicated by an *arrow*. The protein of interest is inserted between *Sal*I and *Mlu*I restriction sites that were engineered into the vector for this purpose (i.e. two two-residue linker sequences, in *green*). The protein of interest is C-terminally fuses to a C-terminal fragment of Lp_2578 including the LPxTG anchoring domain consisting of the LPxTG motif (*red*; the consensus sequence in *L. plantarum* is LPQTxE [148]), followed by a highly hydrophobic stretch (black) and positively charged C-terminal arginine residues (*blue*). The length of the Lp_2578 linker may be varied and so far three variants have been constructed, all of which were shown to work [41]. Full length N-terminally processed and cell-wall anchored Lp_2578 is 647 residues of which the last 194 residues are a region of low complexity. The three available linkers comprise 128, 194 and 644 residues, corresponding to a truncated low-complexity region, the complete low complexity region, and almost the complete protein, respectively

Targeting proteins to the cell wall can be done by fusing the carrier protein to an LPXTG-type cell wall anchor [60, 61]. These C-terminal anchors contain an LPXTG sequence motif followed by hydrophobic amino acids and a short tail of positively charged residues (Fig. 2). LPXTG anchored proteins harbor an N-terminal signal sequence that directs export across the cell membrane via the Sec-pathway. Upon secretion, a transpeptidase called sortase cleaves between Thr and Gly in the LPXTG motif and the protein is covalently attached to the cell wall peptidoglycan via the threonine [62] (Fig. 2). Directly upstream of the LPXTG motif, i.e. between the anchoring point and the catalytic domain of the anchored protein, one usually finds a low complexity linker region of about 50–150 residues which is rich in proline/glycine and/or threonine/serine residues [18]. There are two major differences between sortase-mediated anchoring and the anchoring methods described before: (1) the anchored protein is attached to cell wall which is more peripheral and (2) the protein is attached to the surface via its C-terminus. Literature contains several examples of successful sortase-mediated cell-wall anchoring of heterologous proteins in both *Lactobacillus* and *L. lactis* [20, 41, 63–67].

The *Streptococcus pyogenes* M6 protein has been extensively used for anchoring proteins in LAB [27, 64, 65, 68]. The length of the linker region was shown to be important for both surface exposure and stability of the target protein. In a landmark study, Dieye et al. [27] used the M6 protein as anchor for displaying the NucA reporter. When using anchoring fragments including the complete low-complexity linker region, several NucA-containing protein bands were detected in the cell wall fraction, indicating proteolytic degradation. After deletion of the 105 residue linker region preceding the anchoring motif one major band was found in the cell wall fraction. These authors also showed that NucA containing the linker was efficiently translocated and N-terminally processed, but that sortase processing was slow, possibly resulting in limited anchoring and higher proteolytic susceptibility [27].

As part of the development of the pSIP vector system, Fredriksen et al. [41] used various variants of the LPXTG anchor of Lp_2578, a 705 residue protein annotated as “cell wall surface adherence protein” from *L. plantarum* WCFS1 (Fig. 2). The 37 k Da human oncofetal antigen (OFA) was N-terminally fused to a selected signal peptide (from Lp_0373) and C-terminally fused to N-terminally truncated variants of Lp_2578. The length of the “linker region”, i.e. the length of the Lp_2578 fragment counted from the cell wall anchored threonine and backwards, was 644, 194 or 128 residues (full length N-terminally processed and cell-wall anchored Lp_2578 is 647 residues). The 194 residue linker contains the complete low-complexity region upstream of the LPXTG motif, whereas this region is truncated in the shorter 128 residues variant. In all cases OFA was detected in the cell wall fraction, but the constructs differed in terms of the amount of protein produced, the degree of proteolytic degradation and the amount of OFA observed in the supernatant (indicating shedding and/or limited sortase processing). The differences show that the lengths of the anchors and linker sequences affect anchoring efficiency and proteolytic stability, but that it is as yet difficult to predict the magnitude and direction of such differences. Hence, a modular vector system, such as the pSIP system, allowing rapid testing of various anchors, as well as testing of other factors such as promoter strength and the signal peptide, can be useful.

Available data clearly show that expression of these complex fusion proteins is not straightforward and tends to lead to reduced bacterial fitness and/or proteolytic stress on the displayed protein. Reduction of proteolytic degradation could be obtained by deleting proteases as demonstrated for *L. lactis*, which showed reduced proteolysis of several heterologously expressed antigens and enzymes upon deletion of the HtrA protease [69]. Another variable of interest is the choice of the expression host, since hosts may show varying proteolytic activities as well as varying secretion and anchoring abilities. For example, lactococci generally show stronger proteolytic activity compared to lactobacilli [27]. Proper translocation and subsequent surface location of the target protein also depend on the secretion signal, meaning that the choice of signal peptide is of utmost importance, as alluded to above.

### Non-covalent binding-domain-mediated anchoring

Proteins can be non-covalently attached to the cell wall of gram-positive bacteria by being appended to a variety of cell wall binding domains, including surface layer proteins and surface layer homology domains (SLPs and SLHDs), LysM domains, GW modules, WxL domains, and various other cell wall binding domains, including choline-binding domains, as reviewed in [70, 71]. Notably, whereas several lactobacilli produce SLPs and, thus, S-layers, none seem to contain proteins carrying SLHDs [72].

Several *Lactobacillus* species have S-layers, i.e. a porous two-dimensional lattice of self-assembled proteins varying in size from 40 to 200 k Da that entirely coats the bacterial surface. In some lactobacilli, up to 10–15 % of the total proteins are S-layer proteins, indicating high production and efficient secretion of these proteins. Since S-layer proteins are produced in high amounts, the cognate promoters and/or signal peptides have been exploited for expression and secretion of heterologous proteins in LAB [73–77]. S-layer proteins contain a region that binds strongly to the cell envelope. Another region is involved in self-assembly of the S-layer and is most likely surface exposed [72].

Only few studies have explored the possibility of using SLPs as carrier for surface-display of heterologous proteins in *Lactobacillus* and *Lactococcus*. It has been shown that insertion of short heterologous epitopes in the surface layer sequence results in surface exposure, without significantly affecting S-layer assembly [78, 79]. In another approach, Hu et al. [80] fused the C-terminal region of the SLP of *L. crispatus* K2-4-3 to Green Fluorescent Protein (GFP), and expressed the GFP-SLP hybrid in *E. coli*. The purified fusion protein was shown to bind to the surfaces of several S-layer-free *Lactobacillus* strains as well as *L. lactis*. In addition, the GFP-SLP fusion protein was successfully expressed in *L. lactis*, where anchoring to the surface was achieved. These studies, few as they may be, clearly show the potential of using the SLP anchor for surface display in hosts that do not produce SLPs themselves [80].

Proteins may also be bound non-covalently to the cell surface of LAB by LysM domains. These domains are widespread and may be present in single or multiple copies in a protein. LysM domains are most often located in the N-terminal or the C-terminal part of proteins, and less frequently in a central region. LysM domains vary in length from 44 to 65 residues and bind to peptidoglycan and chitin [18, 81–83]. The peptidoglycan binding properties have been widely used for surface display, as recently reviewed by Visweswaran et al. [84]. The LysM anchor can be used for non-GMO strategies that are based on producing LysM-containing hybrid proteins in a producer strain e.g. *E. coli* or *L. lactis*, followed by charging a suitable LAB with this recombinant protein [85–88]. Alternatively, a hybrid protein consisting of a signal peptide, the LysM domain(s) and the target protein may be expressed directly in lactobacilli or lactococci [21, 89–91]. LysM domains are often separated from each other and from the other domains by linker sequences rich in Ser-, Asn-, and Thr residues. When designing LysM containing hybrid proteins for surface display, these linker regions should be considered to be included in the hybrid protein. The linker region may convey flexibility and mobility to the fused target protein, which may allow this protein to attain an optimal orientation in the cell wall.

Importantly, as shown in the work referred to above, the LysM anchoring motif can also be exploited for developing GMO-free carrier systems, by over-expressing the LysM-containing hybrid protein in one expression strain followed by immobilization onto another non-recombinant host, e.g. a LAB [85–88]. The LysM-containing hybrid protein can be purified using conventional easy-to-use methods, e.g. by using a N- or C-terminal purification tag, or a crude protein extract can be mixed with the carrier host [85]. Notably, compared to recombinant production, this approach allows easier control over the amount of protein loaded onto the cell surface. A non-GMO strategy not depending on LysM domains was described by Brinster et al. in 2007 who showed that a hybrid protein of NucA fused to a WxL domain from *Enterococcus faecalis* bound to several gram-positive bacteria, including *L. johnsonii* [92]. In this study, it was convincingly shown that the binding target for the hybrid protein was peptidoglycan of the cell wall.

## Application of lactic acid bacteria for displaying proteins

**Fig. 3:**
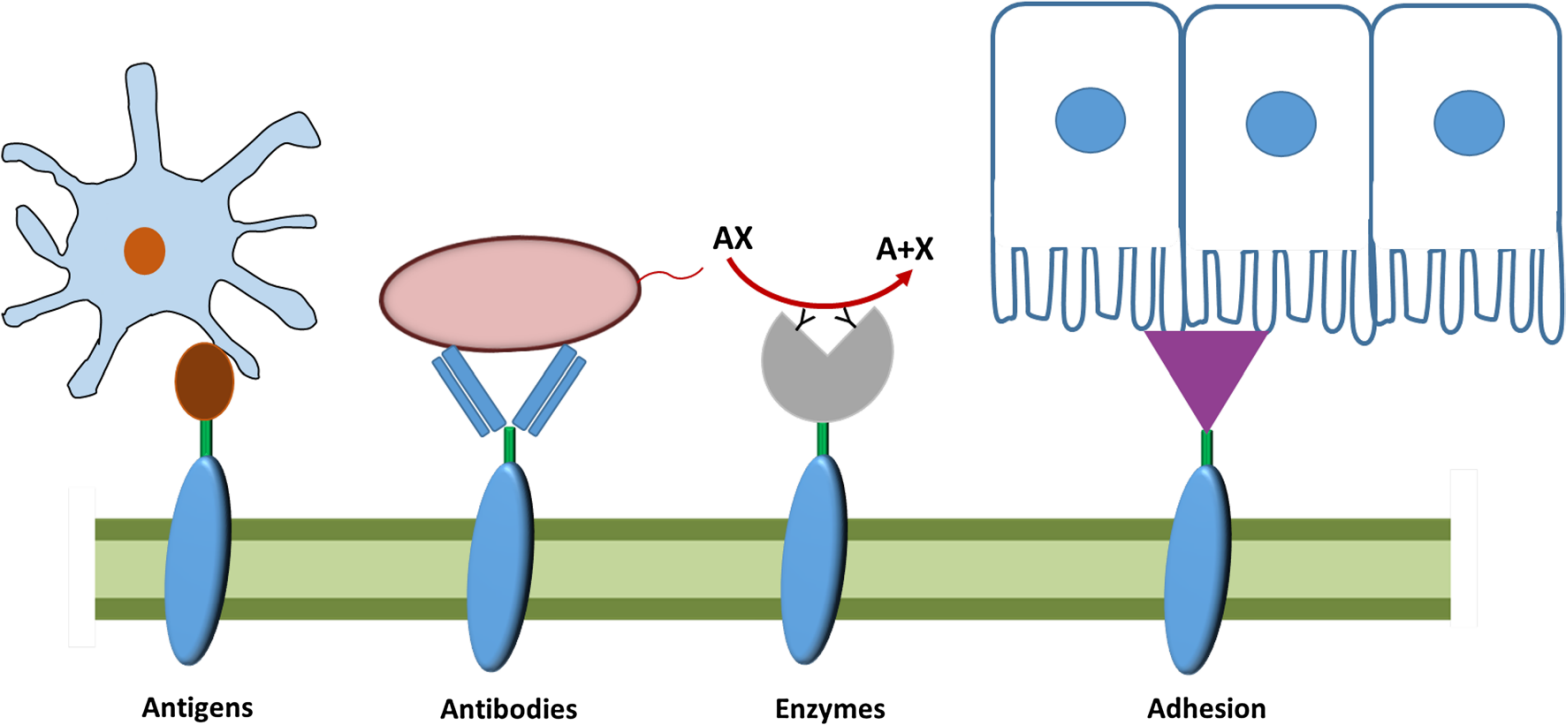
Global view of applications of protein display at surface of LAB. Antigen display at surface of LAB can be used for various goals as: increasing the immune response (antigen display; adhesive protein display; antibodies display), metabolic engineering (enzyme display) or provide a passive immune response (antibodies display)

The remainder of this review will address various applications of the anchoring strategies discussed above, and will address the potential advantages of the anchoring approach compared to other protein delivery strategies such as secretion or cytosolic production. Figure 3 provides a schematic overview of possible applications of protein-displaying LAB.

### Quantification of surface display

It is important to note that studies on protein surface display in LAB or more generally heterologous protein expression are usually not quantitative. Quantitative informations may be obtained using semi-quantitative analytical tools such as western blotting, flow cytometry or fluorescence microscopy. Indeed within single studies, using the same analytical tools to compare different engineered strains, some degree of relative quantification can be obtained. Consequently, in many of the examples discussed below, differences observed between different strains and anchoring techniques may relate not only to the localization and stability of the expressed protein, but also to the level of protein expression.

### LAB displaying antigens

In the last decade several studies have successfully shown delivery of different kinds of antigens by LAB. The targeted pathogens include HPV-16, avian influenza viruses and different kinds of parasites [3]. One of the strategies used is the display of antigens at the surface of LAB. Pioneering work in the field concerned production of the tetanus toxin from *Clostridium tetani* in *L. lactis*. This toxin has been regularly used as a model antigen, for example in comparative studies of strains targeting the toxin to different locations. In 1997, Norton et al. expressed the tetanus toxin fragment C (TTFC) in *L. lactis* [6] and showed that intranasal administration of the bacteria mice elicited a specific immune response reflected in production of both serum and secreted specific antibodies. In a second study, the TTFC was expressed in *L. plantarum* and directed to all three possible locations: surface-exposed (using the M6 anchor), secreted or cytoplasmic [93]. All three types of localization led to specific immune responses. Protein quantification is a challenging aspect in this type of studies. In this pioneering work, it was noted that the highest IgG serum antibody titers were obtained with the strain producing the largest amounts of TTFC in the cytoplasm. This led the authors to suggest that the intensity of the response is mainly driven by the quantity of the produced antigen.

The most detailed study on LAB-based vaccines concerns vaccination against human papillomavirus-16 (HPV). HPV is the causative agent of cervical cancer and oncoprotein E7 produced in tumor cells is known to be an efficient antigen [94]. There exist numerous studies on the development of vaccines against HPV that are based on using E7 [95–97]. E7 has been successfully displayed on the surface of *L. lactis* [98] and intranasal immunization of C57BL/6 mice with the recombinant bacteria induced an E7 specific immune response [67]. Optimization of the expression system, by using a nisin-induced promoter to control E7 expression, led to enhanced E7 production and a better immune response, which clearly showed that the antigen level is important for effectiveness [14]. In a follow-up study, a *L. lactis* strain displaying E7 on its surface was co-administered with *L. lactis* secreting pro-inflammatory interleukin 12 [99], leading to a strongly increased immune response. A strain co-expressing both proteins showed therapeutic effects on HPV-16-induced tumors in mice [100]. Building on these promising results Ribelles et al. recently explored the possibility to generate a non-genetically modified LAB displaying the oncoprotein. To achieve this, E7 fused to a His-tag (for protein purification) and the cell wall binding domain from the A2 phage lysin of *Lactobacillus casei* was produced in *E. coli*, purified and attached to the surface of (non-recombinant) *L. lactis* or *L. casei* [101]. Administration of these strains did elicit an immune response in C57BL/6 mice, but the response was weaker compared to responses seen for the genetically-modified strains.

A priori, one would expect that the localization of the antigen in the expression host affects the immune response. Intracellular localization may protect the antigen from the harsh conditions in the GIT, however the antigen will only be accessible for interactions with the target cells after cell lysis. Secreted and surface-anchored proteins are more prone to degradation, but also more accessible to host cells. Comparison of immune responses elicited in mice by various recombinant LAB expressing E7 showed that the higher antigen-specific cellular response was obtained after nasal administration of LAB producing cell wall anchored E7 compared to LAB producing intracellular or secreted E7 [14]. The authors hypothesized that proteoglycan compounds found in the cell wall could act as adjuvant, thus potentially strengthening the response elicited by surface-displayed E7. Supporting a possible role of cell wall components, it has been shown that the probiotic properties of *L. salivarius* Ls33 are driven by components in the cell wall, in particular a peptidoglycan-derived muropeptide [102]. This study, as well as other studies cited below do clearly show that localization plays a role, but do not reveal a general trend as to what type of localization is generally most promising.

Interestingly, lactobacilli expressing surface-anchored E7 are one of the few strains which have been evaluated in a human clinical study. The strain was used as therapeutic vaccines in a single-center, single-arm (non-controlled), observational phase I/IIa study. It was shown that, after oral administration, heat inactivated *L. casei* producing the E7 antigen with a PgsA N-terminal transmembrane helix anchor elicits an E7-specific mucosal immune response, but not systemic cell-mediated immune response [12]. The treatment was safe and adverse effects on patients were not observed. This study thus showed that heat attenuated recombinant lactobacilli can induce an immune response in humans. This is an interesting result because there is an ongoing discussion on the importance of using live bacteria rather than non-viable cells. In another study, oral immunization with the heat inactivated strain was shown to elicit E7-specific IFN-γ-producing cells in mice [54].

Rotaviruses are the main cause of diarrhea in children around the world. It has been shown that *L. lactis* with surface displayed rotavirus VP8 antigen using the M6 cell wall anchor, induced secretion of antigen specific antibodies in mice, both in the intestine and at the systemic level. *L. lactis* producing VP8* intracellularly induced only mucosal secretion of IgA [103], suggesting that surface exposed localization of the antigen is most efficient. In another study, it was shown that *L. lactis* strain secreting VP7 elicited production of neutralizing antibodies after oral administration of the recombinant bacteria to mice. In contrast to other studies, in this case, strains expressing the cell wall anchored antigen did not elicit an immune response. The authors suggested that in this case no adjuvant effect was provided by other components of the *Lactococcus* cell wall, while the efficiency of the intracellular localization was explained by lysis of the bacteria [104]. It was shown that the VP7 protein was degraded, which may imply that the protein is sensitive to the conditions in the GIT, which again could explain why surface localization was not efficient. This study underpins two important aspects of LAB-based vaccine delivery: (1) the final localization of the target antigen has great impact on the immune response and (2) while surface-display a priori seems promising, it is not necessarily so that surface-display is the method of choice in all situations.

Hugentobler and colleagues have engineered *L. lactis* strains expressing the *Leishmania* major antigen LACK in the cytoplasm, in a secreted form or with a cell wall anchor, with or without concomitant IL-12 secretion. After oral administration, only the strain secreting the antigen and co-expressing IL-12 induced protection against the parasite [105]. In another study these same authors showed that subcutaneous co-administration of *L. lactis* secreting IL-12 with either a strain secreting LACK or a strain with a cell wall anchored (CWA)-LACK led to induction of protective immunity [106]. The lack of effectiveness of the CWA-LACK strain in oral immunization could be due to the fact that the expression strain was an *alr* mutant, which could lead to impaired cell-wall strength and, possibly, antigen anchoring.

In an attempt to develop a protective agent against *Giardia lambia*, the cyst wall protein 2 from the parasite was produced in *L. lactis*, either in the cytoplasm, displayed at the surface or secreted. Mice trials were done with the strain harboring cell wall anchored antigen which elicited a specific mucosal IgA antibody response [107]. Importantly, challenge experiments showed that mice immunized with the recombinant strain were partly protected from infection.

### LAB displaying antibodies

Immunization strategies based on antigen delivery require a strong response from the host immune system. Alternatively, antibodies produced by LAB can be used to generate passive immunity, thus providing a more direct method, with fast response time.

In a landmark study, Krüger et al. [30] engineered *L. zeae* strains producing a single-chain antibody fragment (ScFv) targeting the SAI/II antigen from *Streptococcus mutans*. They explored secreted ScFv as well as cell-wall anchored ScFv with either a short or a long anchoring sequence. Production of a functional ScFv fragment was confirmed for all strains. In agglutination tests, only the strain producing ScFv with the long anchoring fragment showed good efficiency. This latter strain was also tested in a rat model of dental caries development. After administration of the ScFv producing strain, both dental caries and the number of oral *S. mutans* were markedly reduced [30]. This was the first demonstration of the possibility of using LAB for generation of effective passive immunity.

*Porphyromonas gingivali*, is one of the major etiologic agents of periodontitis, an inflammation of the tissue surrounding the teeth [108]. A functional ScFv targeting the RgpA protease from *P. gingivali* has been successfully produced and surface displayed in *L. paracasei* using a long anchor sequence [109]. The ScFv expressing strains were able to agglutinate to *P. gingivali*.

Building on these successes in expressing ScFv’s more recent studies have explored the potential use of LAB in handling the threats of bioterrorism. The protective antigen (PA) from *Bacillus anthracis* is a perfect target for efforts to neutralize pathogenicity [110]. In a recent study, an anti-PA ScFv was secreted or cell wall anchored in *L. paracasei*, using an LPxTG anchor or non-covalent anchoring through an SLP-like domain. In vitro studies showed that when macrophages were exposed to a lethal dose of toxin, addition of *L. paracasei* secreting or displaying the ScFv gave almost full protection. In vivo experiments with mice showed that *L. paracasei* displaying the ScFv at the surface through non-covalent attachment induced protection, whereas strains secreting the ScFv or with the ScFv covalently anchored to the surface did not. The authors suggested that, in non-covalent attachment, the ScFv is both cell wall displayed and secreted into the supernatant, which may have been beneficial in their experimental set-up [111].

Infliximab^®^, one of the most efficient drugs against inflammatory bowel diseases (IBD), is a recombinant antibody targeting TNF-α, a major pro-inflammatory cytokine. An effective recombinant affibody targeting TNF-α composed of an affinity domain for TNF-α and a peptidoglycan binding domain, AcmA, has been successfully produced and displayed at the surface of *L. lactis* [91]. The authors of this study do not report any test of efficiency of the strain in vitro or in vivo. However, another team has shown that a recombinant strain of *L. lactis* secreting MT1, a nanobody binding to TNF-α, has a beneficial effect on dextran sodium sulfate (DSS)-induced colitis and IL-10 knock-out mice [112]. In the latter study, it was shown that only a small quantity of antibody was needed to induce protection, and that protection occurred even in the very early stage of the development of colitis.

### Display of proteins to increase interactions between LAB and host cells

In order to obtain strong responses to LAB expressing therapeutic molecules it may be beneficial to modify the bacteria with factors that increase their interactions with appropriate host cells. Therefore, several studies have explored the potential of adding such factors to LAB, be it separately expressed adhesive factors, such as fibronectin-binding protein, or factors fused to the therapeutic protein.

Fibronectin-binding protein A (FnBPA) is a surface-located adhesin and virulence factor from *S. aureus*, which enables the bacterium to adhere to host cells and facilitates bacterial internalization. FnBPA binds to fibronectin, which is produced in the extracellular matrix of various eukaryotic cells [113]. Another protein that promotes binding and internalization is Internalin A (InlA) from *Listeria monocytogenes.* InlA binds specifically to human E-cadherin, which is present in epithelial cells [114], but has low affinity for its murine counterpart [115]. A mutated form of InlA (mInIA) has been developed which has increased affinity for murine E-cadherin [115–117].

Both FnBPA and mInlA have been successfully expressed and displayed at the surface of *L. lactis*. Furthermore, it has been shown that lactococci harboring mInlA or FnBPA at the surface were more efficiently internalized by caco-2 cells, compared to the wild-type strains [118, 119]. In these studies surface display was achieved by using LPXTG domain anchors.

Wild type lactococci are naturally able to transfer plasmids to eukaryotic cells at a low rate [119]. This offers a potential for gene therapy. The transferred plasmid can be used for delivery of a cDNA containing a target gene under transcriptional control of an eukaryotic promoter, such as the human cytomegalovirus (CMV) promoter, to elicit expression of a protein of interest by the host cells. Using gene transfer for monitoring internalization, it has been shown that expression of FnBPA or mInlA at the surface of *L. lactis* increases bacterial internalization in vitro and in vivo in mice. Successful expression of genes transferred to eukaryotic cells has been demonstrated with different proteins, including the major milk allergen, beta-lactoglobulin, GFP or IL-10 [116, 120, 121]. These initial studies clearly show the potential of using LAB for the delivery of cDNA for subsequent intracellular production of medically interesting proteins in the host cells.

In another study, also aiming at gene transfer, lactobacilli were made to express an ScFv called aDec that targets Dec205, a receptor on the surface of dendritic cells (DCs) [122]. Three anchoring strategies were used: covalent anchoring to the cell membrane (Lipobox), covalent anchoring to the cell wall through an LPXTG anchor (CWA) and non-covalent anchoring to the cell wall using a LysM domain (LysM). This study revealed clear differences between the anchoring strategies. Surface location of the antibody could only be demonstrated for the strains with cell wall anchors (CWA and LysM) and only these two strains showed increased uptake into DCs in vitro, as well as increased plasmid transfer from the lactobacilli to DCs. Interestingly, in mice experiments, the highest plasmid transfer was observed for the strain in which aDec was coupled to the cell membrane (Lipobox). It is conceivable that in the case of Lipobox anchoring aDec is more embedded in the cell wall and thus better protected from the harsh conditions in the GIT of the mice.

Another illustration of the functional implications of different anchoring strategies comes from the studies on expression of the invasin (Inv) of *Y. pseudotuberculosis* in lactobacilli. Inv was displayed at the surface using LPXTG-based covalent binding, or via fusion to LysM or lipoanchor domains. In vitro experiments with U937 cells stably transfected with the NF-κB reporter plasmid 3x-κB-luc [123] showed that the most effective NF-κB response was obtained with constructs in which the complete Inv extracellular domain was fused to a lipoanchor [21].

Microfold cells (M-cells) are found at the top of the Peyer’s patches in the epithelial membrane of the small intestine. They sample antigens and bacteria from the lumen and mediate subsequent transfer to the Peyer’s patches, via a process called transcytosis. Sigma C is a protein from avian reovirus that binds specifically to M-cells and which could thus be used for targeting these cells. Lin et al. explored this option by fusing both the Sigma C protein and a sub-fragment of the spike protein from the avian Infectious Bronchitis Virus, a known antigen for vaccination purposes, to a LysM domain derived from lactococcal AcmA. After production of the two proteins in *E. coli* and subsequent purification using a His-tag, they charged a non-pathogenic *Enterococcus faecium* with one or both of the LysM-anchored proteins. *In vitro* and in vivo (mice) experiments showed that enterococcal cells carrying both proteins elicited a better immune response against the virus than bacteria charged with the spike sub-fragment only [124]. Thus, targeting of M-cells seems to be a promising strategy to enhance immune responses.

Targeting dendritic cells, e.g. by targeting the Dec205 receptor described above, is generally considered highly promising in vaccine development and there are many examples in the literature, including several examples involving the targeting of LAB [125]. One of the most promising studies in the field *Lactobacillus*-based vaccines concerns the development of lactobacilli capable of protecting mice against *B. anthracis* [126, 127]. In this study, *L. acidophilus* and *L. gasseri* strains secreting the *B.anthracis* PA were developed. The key of the success was to fuse the PA to a peptide with known high affinity for DCs [128]. Apart from revealing protective effects of a LAB-based vaccine, this pioneering work from the Klaenhammer team showed that targeting the antigen-displaying LAB to DCs has a strong positive effect on immunostimulation. DC-binding peptides are currently receiving considerable attention, including the discovery of novel peptide sequences [125, 129]. Recent studies, as well as our own as yet unpublished data confirm the usefulness of adding a DC-binding peptide sequence to antigens displayed on LAB-surfaces [130].

### Enzyme display and metabolic engineering

LAB have the ability to metabolize various nutrients, including a range of pentose and hexose sugars. Therefore, there is increasing interest in developing LAB as cell factories for industrial enzymes and for use in consolidated biomass bioconversion processes [131]. Although LAB are not known as high-efficiency secretion hosts, their ability to simultaneously secrete and/or anchor several enzymes and their robustness do make them interesting candidates for such purposes. A potential limitation concerns the fact that the main product of fermentation is lactic acid. While lactic acid has considerable value, rerouting of metabolic pathways will be needed if other products are to be made, such as ethanol [132].

Promising results have been obtained in endorsing LAB with amylolytic capacity, mainly through making bacteria secreting amylases [131]. For example, Narita et al. used the N-terminal transmembrane PgsA anchor to display α-amylase from *S. bovis* on the surface of *L. casei.* They showed that the recombinant strain was capable of converting soluble starch to lactic acid [33]. This study was one of the first to show the feasibility of displaying active recombinant enzymes on the surface of LAB.

Conversion of lignocellulose requires multiple enzymes with different substrate specificities. Literature contains a number of studies describing the expression and secretion of individual cellulases in LAB [137], which certainly is not enough for efficient biomass conversion. In anaerobic bacteria, the various enzymes are organized in cellulosomes, i.e. protein complexes containing a multitude of enzymes [133].

**Fig. 4:**
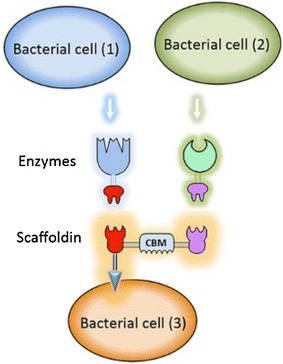
Display of a mini-cellulosome. The picture, taken from Morais et al. [139], BfB, shows how a consortium of recombinant lactobacilli may be used to create LAB displaying a mini-cellulosome. Two strains produce one secreted glycoside hydrolase each; the enzymes are fused to dockerin domains. A third strain produces a scaffoldin that is anchored to the cell surface. The secreted glycoside hydrolases will dock onto the scaffoldin through their dockerin domains, thus forming a mini-cellulosome

In a cellulosome, multiple enzymes containing so-called dockerin domains are assembled on a scaffold, which is often anchored to the cell surface and containing so-called cohesion domains that bind the dockerins. One strategy would be to make LAB producing mini-cellulosomes, i.e. cellulosomes comprising a small scaffold and only a few enzymes [134]. Such a strategy has been successfully applied to yeast [135, 136] but in LAB only some first steps into this direction, have been described [65, 137]. In particular, it has been shown that a scaffold, anchored to the surface of *L. lactis* using an LPxTG anchor, binds specifically to enzymes containing a dockerin domain and that the bound enzymes are active [65, 137] (Fig. 4). By analogy to the cellulosome paradigm, it would be beneficial if the LAB bind to the lignocellulose substrate. This has been achieved for *L. lactis* by surface-display of the cellulose-binding domain of XylA, a xylanase from *Cellvibrio japonicas* using a LPXTG or a LysM anchor [138].

In a very recent study, Morais et al. used the versatility of the pSIP expression system to produce what could be called the first functional minicellulosome in LAB [139]. In one part of this study, strains producing cell-wall anchored variants of the previously used xylanase and cellulase were generated using previously developed LPxTG anchors [41]. In another part of the study, the carbohydrate-binding modules of the (non-anchored) cellulase and xylanase were replaced by dockerins, whereas a third strain was engineered that expressed an LPxTG-anchored scaffoldin with two cohesin domains (i.e. docking positions for dockerins) and a carbohydrate-binding module. The mix of the three strains led to assembly of a minicellulosome and resulted in wheat straw degrading activity similar to the one observed when mixing the two individual enzyme-producing strains.

## Conclusions

Taken together, the accumulated results of in vivo studies show that while it is not straightforward to predict the best localization of the target protein, surface-display tends to be a good strategy for reasons that are likely related to the accessibility and stability of the displayed protein. Importantly, the studies described above are by and large non-quantitative, meaning that it remains mostly unclear whether functional differences between different strategies simply relate to the amount of protein expressed rather than to the localization and stability of the expressed protein. There is a clear need for approaches with more focus on quantification, to discriminate between localization effects and dose–response effects. Interesting open questions that relate to the quantification issue concern the potentially interrelated effects of the anchoring-type on in vivo stability and proteolytic susceptibility of the displayed protein and on protein accessibility for host cells. Varying anchoring strategies will lead to varying degrees of protein shedding, which also may have (quantitative) effects on strain functionality.

High production levels of the to-be-displayed protein may seem beneficial and may be achieved by using strong inducible or constitutive promoters, as well as by codon optimization of the to-be-expressed gene. However, unbalance in the transcription-translation-secretion machinery, caused by e.g. too high transcription levels or suboptimal translation rates may cause problems, leading to retarded growth of the producer strain and/or to secretion stress, or leading to reduced secretion efficiency and proteolytic degradation of the target protein. The choice of signal peptide affects also the secretion efficiency. Signal peptide performance shows considerable variation and is partly dependent on the target protein [23]. Unfortunately, signal peptide performance and secretion efficiencies are notoriously difficult to predict, meaning that the best strategy for strain development is to use an expression vector system that allows rapid screening of numerous variants. Clearly, deeper functional insight into the factors that govern the efficiency of the secretion of heterologous proteins in LAB would be highly useful.

Rapid screening of various expression constructs should also allow variation of the anchors since the data accumulated so far do not pinpoint to one particular type of localization or anchoring mode as superior. Depending on its localization, the translocated recombinant protein will be exposed to the harsh conditions in the GIT and one would expect a trade-off between being vulnerable for proteolysis and being visible to the immune system of the host. Unanswered questions include: Will membrane-anchored proteins be more protected from the harsh conditions in the GIT, compared to cell wall anchored proteins? Which significance has the length of the linker between the cell wall-anchoring motif and the recombinant protein?

When it comes to application of the engineered LAB, one open question is whether live and replicating or non-viable bacteria should be used. Clinical studies have shown that both attenuated [12] and living bacteria are promising [140] delivery vehicles. In replicating bacteria, it is important that the promoter is active in situ, otherwise recombinant surface located proteins will be diluted from the surface, which will likely result in a reduced immune stimulatory effect. Promoters whose expression is induced during passage through the GIT of mice [141] may be good candidates for in situ expression.

To date, several recombinant LAB have reached clinical studies [11]. The first clinical study was completed in 2006 using *L. lactis* for secretion of human IL-10 in patients with Crohn’s disease [142]. Ten patients were included in a placebo-uncontrolled phase I trial. The treatment was shown to be safe and was well tolerated by the patients, while Crohn’s disease symptoms were reduced. However, the beneficial effects of this engineered *L. lactis* strain could not be confirmed in a following phase II clinical study. Another more recent study concerned *L. lactis* secreting trefoil factor (TFF), which is an anti-inflammatory protein. It has been shown that TFFs reduce the severity of radiation-induced oral mucositis when administrated in hamsters [143]. In a phase 1b, single blinded, placebo-controlled experiment, the patient received recombinant *L. lactis* secreting TFF 1(AG013) orally. The results demonstrated that AG013 was safe and well tolerated, and reduced oral mucositis [140]. A phase II study has been designed to further validate the efficacy of AG013. The last known example is an ongoing clinical study using a recombinant *L.lactis*, AG014, which secretes anti-TNF-alpha Fab to treat inflammatory bowel disease. An example of clinical studies with *L. casei* has been described above [12].

Much of the pioneering work in this area was done using *L. lactis*, a well-known bacterium in dairy products which does not survive in the GIT more than a few hours [2]. In contrast, some lactobacilli survive the GIT; they can reside in the body for several days and some of them are even considered commensal, meaning that they are part of our intestinal microbiota. Many have been described with beneficial effects [144, 145]. It has to be noted that the genetic toolbox for lactobacilli is well developed and that many of the most recent and most promising studies on functional display of proteins in LAB concern lactobacilli.

Further insight into lactobacilli is emerging from massive genomics efforts, while the emergence of CRISPR-Cas technologies provides novel genetic engineering tools of unprecedented quality, safety and precision [146, 147]. These bacteria, with their ecological versatility, robustness and known beneficial effects, are likely to become major players in further development of LAB-based vaccines and cell factories.

## Abbreviations

LAB: lactic acid bacteria
GIT: gastro-intestinal tract
WHO: World Health Organization
SP: signal peptide
NucA: staphylococcal nuclease reporter protein
BmpA: basic membrane protein A
LPS: lipopolysaccharides
OFA: oncofetal antigen
SLPs: surface layer proteins
SLHDs: surface layer homology domains
GFP: green fluorescent protein
TTFC: tetanus toxin fragment C
HPV: human papillomavirus-16
CWA: cell wall anchored
ScFv: single-chain antibody fragment
PA: protective antigen
IBD: inflammatory bowel diseases
DSS: dextran sodium sulfate
FnBPA: fibronectin-binding protein A
InlA: internalin A
mInIA: mutated form of InlA
CMV: human cytomegalovirus
DCs: dendritic cells
Inv: invasin of *Yersinia pseudotuberculosis*
M-cells: microfold cells
